# Humans Conceptualize Victory and Defeat in Body Size

**DOI:** 10.1038/srep44136

**Published:** 2017-03-15

**Authors:** Wenjun Yu, Zhongqiang Sun, Jifan Zhou, Chaoer Xu, Mowei Shen

**Affiliations:** 1 Zhejiang University, Department of Psychology and Behavioural Sciences, Hangzhou, 310027, China; 2 Ningbo University, Department of Psychology, Ningbo, 315000, China

## Abstract

Size matters considerably for victory and defeat during competitive situations. Drawing on the embodied theory of cognition, we examined the reciprocal association between size and competition outcomes. To do so, we used the ‘rock-paper-scissors game’, whose outcome is not contingent on apparent physical size. In Experiment 1, participants were asked to judge whether the target gesture was a winning or a losing one. Compared to responses in the incompatible condition (small-winner and large-loser), those in the compatible condition (large-winner and small-loser) were quicker. In Experiment 2, we asked participants to adjust the size of gestures to correspond to gestures previously presented, and found that the winning gesture was estimated as much larger than the losing one. In line with our main hypothesis, size information can interfere with judgments about competition outcomes, and vice versa, even when the outcome is unrelated to body size.

Competition is a ubiquitous behaviour pattern, and, in most cases, it is accompanied by victory and defeat, which can determine an individual’s survival. In daily communication, size is a common theme in relation to victory and defeat: for instance, a conqueror who wins a battle might be called ‘strong and great’, whereas the loser would be called ‘weak and small’. Herein, we empirically investigated whether mental representations of victory and defeat relate to body size.

Most of us learn that size is an important factor for achieving a good result during some competitions. For example, individuals with comparatively large body sizes are more likely to hit smaller people^1^. This phenomenon occurs among adolescents and children^2^. After repeated defeats, children may learn that it is difficult to win competitions when facing adults or larger siblings. Over their ontogenetic development, these recurrent experiences may help reinforce the association between size and competition outcomes.

This ontogenetic phenomenon may also have a phylogenetic basis. Specifically, the association of size and winning may be more common among reptiles and mammals, where physical fights have traditionally been the only way of determining victory. As Freedman^3^ noted, ‘throughout nature the rule is the bigger, the more dangerous’. When exposed to danger, creatures, even smaller ones, tend to crane their necks, enlarge their bodies, and increase their apparent size as a bluff, in order to appear more threatening and thereby deter predators. Additionally, after achieving victory, humans and other mammals tend to stretch out their arms and legs as part of their celebration or expression of excitement^4^,^5^. Taken together, it is not rare to see that the winner of a fight has a large body, suggesting that physical size may be a phylogenetic determinant of competition outcomes.

In the literature on human competition, some recent studies have investigated the relationship between fighting capacity and estimation of physical size. In particular, Fessler and colleagues^6^ introduced the notion of size-as-representation, which suggests that mental representations of prospective foes change with cues signalling potential harm. In their seminal research, an individual’s envisioned physical formidability, as represented by size and strength, can be influenced by access to weaponry^6^, the presence of male companions^7^, and the leadership effectiveness of enemy commanders^8^, etc. Besides, such relationship between physical size and capacity would further affect individual’s decision-making process^9^,^10^.

With the increasingly complex competition situations in current society, physical size no longer has direct relevance to competition outcome. Instead, multimodal characteristics such as intelligence, emotion quotient, or even luck have become the major determinants of achieving victory. In this case, the phenomenal association of size and competition outcome should be reconsidered. Specifically, is it merely common to *see* this association during physical fighting, or has it been schematized in the conceptual architecture of victory and defeat?

Barsalou’s perceptual symbol theory (PST)^10^, which highlights the embodied nature of representations, might provide a theoretical basis for answering the above question. PST proposes that our view of the world is determined by experiences such as body structure and sensorimotor information. Advocates of PST tend to explain humans’ higher-order cognitive processes in terms of modal perceptual symbols, which are derived from multiple sources of direct experience and used to simulate or re-enact a perceptual, acted, or introspected state. Similarly, experimental research has shown that other attributes of formidability, such as position in a social hierarchy, are also associated with size information^11^,^12^. Thus, drawing on both theoretical and experimental underpinnings, body size can be inferred as a core dimension of mental representations of victory and defeat.

Based on empirical evidence, we adopted ‘winner’ and ‘loser’ as the agents of victory and defeat, respectively, and predicted that the direct physical experience of size differences have been schematized into the perceptual symbols of winner and loser—in other words, there are strong mental associations of large size with winner and small size with loser—even when competition outcomes are only dominated by luck. If so, then individuals’ cognitions about winners and losers should be consistently influenced by size information in such luck-based situations.

We tested this hypothesis using a rock-paper-scissors (RPS) game, which is widely adopted in competition studies^13^,^14^,^15^. In this simple and popular game, each gesture can be a winning or a losing one, depending on the pair (i.e., rock defeats scissors, scissors defeats paper, and paper defeats rock); thus, each gesture is mutually restricted, which serves as a natural counterbalancing procedure to minimize the possible influence of their visual differences. By filtering meaningless noise information, the core components—the contestants and game results—can be clearly extracted. More importantly, unlike most conflicts involving physical violence, where the results can be predicted by the physical size difference between the two parties to some extent, the outcome of the RPS game is luck-based—apparent physical size or strength are irrelevant to the outcome. As such, we can use it to investigate whether the mind involuntarily associates size information with the concepts of victory and defeat.

Our first aim was to demonstrate whether size information interferes with judgments of competition outcome. To do this, we employed the interference paradigm, which has proven to be a valuable tool for testing embodiment^16^,^17^. In this paradigm, a Stroop-like interference effect can be observed when the perceptual input is incompatible with the schematized information. We assumed that the compatible content would be ‘winner as large’ and ‘loser as small’ (and thus incompatible content would be the opposite), and asked participants to judge the outcome of the RPS game. If the gesture’s size activated the hypothesized schema, then the judgment speed of competition outcome would be quicker for compatible content compared to incompatible content.

Our second aim was to determine whether the competition outcome would influence the size estimation of gestures. Participants had to recall by adjusting the size of a gesture to correspond with that of the same gesture viewed in a previous RPS game. If activation of the winner or loser schema influenced size estimation, the winning gesture would be adjusted to appear as much larger than the losing gesture. If the above two aims are achieved, we can infer that there is a two-way interaction between mental representations of competition outcome and size.

## Experiment 1

Experiment 1 sought to examine the interference of size information on judgments of competition outcome by manipulating the relative sizes of gesture pairs.

## Methods

### Participants

Thirty-four graduate and undergraduate students (21 females; mean age 21 years) participated in this experiment. None had a history of neurological problems and all had normal or corrected-to-normal vision. The participants provided their written informed consent before the experiment, and were rewarded with course credits or ¥20. All experimental procedures were approved by the Research Ethics Board of Zhejiang University, and were performed in accordance with the approved guidelines.

Given a large effect size (*f* = 0.40; partial *η*^*2*^ = 0.14), a power of 0.80, and an alpha level of 0.05, we found that a between-subjects contrast would be less powerful than both a within-subjects contrast and an interaction. Thus, we used the between-subjects contrast in consideration of the power. The power analysis^18^ ultimately yielded an estimated sample size of 34, so we evenly allocated 17 participants to each between-subjects condition.

### Stimuli

**Figure 1:**
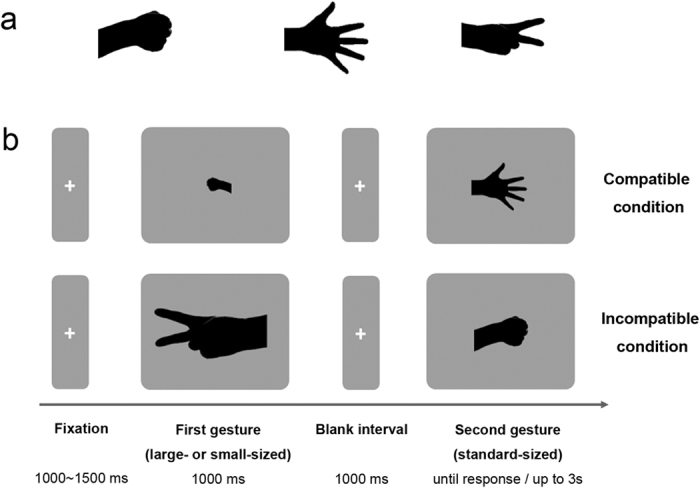
Stimuli and procedure in Experiment 1. (**a**) Three gestures used as experimental stimuli. From left to right, the gestures are rock, paper, and scissors. (**b**) Examples of the procedure. The upper and lower sets of pictures represent Compatible and Incompatible conditions, respectively.

Three pictures of gestures from the RPS game were adopted as experimental stimuli (see Fig. 1). To eliminate the influence of differences in luminance, the gesture pictures were monochromatized to black (RGB: 0, 0, 0). Stimuli were presented on a grey background (RGB: 80, 80, 80) on the CRT monitor of a 17-inch computer (100 Hz refresh rate). Each gesture occupied a rectangular area of approximately 1.4° × 1.2° (small size), 4.4° × 3.8° (standard size), or 7.5° × 6.5° (large size) of visual angle, and was positioned at the centre of the screen.

### Design and Procedure

Participants were seated in an electrically shielded and sound-attenuated recording chamber approximately 70 cm from the CRT monitor. They were asked to keep their eyes centrally fixated.

The experimental design comprised three factors: Judgment (a within-subjects factor, with levels of Win- and Lose-judgment), Compatibility (a within-subjects factor, with levels of Compatible and Incompatible), and Target Position (a between-subjects factor, with levels of Test-in-first and Test-in-second).

The full study procedure is illustrated in Fig. 1. At the beginning of the experiment, half of the participants were told that the first-displayed gesture was the target (Test-in-first condition), and the other half was told the second-displayed gesture was the target (Test-in-second condition). Each trial began with a central fixation cross presented randomly for a duration of 1,000 to 1,500 ms. Subsequently, a small or large sized gesture was displayed for 1,000 ms. After a 1,000-ms blank interval, a standard sized gesture was presented, and participants were asked to indicate whether the target gesture was the winner (Win-judgment condition) or the loser (Lose-judgment condition) among the two sequentially displayed gestures. Responses were made by pressing one of two keys on a keyboard. In the Compatible condition, a large-sized winner was always paired with a standard-sized loser, and a standard-sized winner was always paired with a small-sized loser. In contrast, in the Incompatible condition, a standard-sized winner was always paired with a large-sized loser, and a small-sized winner was always paired with a standard-sized loser.

Each participant in both of the Target Position groups completed 120 trials in total, with 30 trials being evenly distributed among the three possible winner–loser situations (i.e., 10 trials each for Rock–Scissors, Scissors–Paper, and Paper–Rock) for each within-subjects condition. Furthermore, the first-displayed gestures were small sized in 50% of trials and large sized in the remaining 50%. The whole experiment was divided into four sessions with a 2-minute break between sessions. Before the formal experiment, participants were given 15 practice trials to ensure that they understood the instructions.

All results with response times (RTs) either lower than 100 ms or exceeding two standard deviations from the mean were excluded from further analyses. We conducted 2 (Judgment) ×2 (Compatibility) ×2 (Target Position) repeated-measure analyses of variance (ANOVA) on accuracies and RTs for correct answers. The significant main effects were always followed by post-hoc contrasts with a Bonferroni-corrected *p*-value.

## Results

All of participants’ accuracies exceeded 94%, likely because of the task’s high simplicity. The three-way ANOVA for accuracy revealed no significant main effects between the variables: Target Position, *F* (1, 16) = 2.03, *p* = 0.17, *η*_*p*_^*2*^ = 0.11; Judgment, *F* (1, 16) = 0.14, *p* > 0.250, *η*_*p*_^*2*^ = 0.01; Compatibility, *F* (1, 16) = 1.19, *p* > 0.25, *η*_*p*_^*2*^ = 0.07. Neither the two-way, *p*s > 0.250, nor the three-way interaction were significant, *p* = 0.07.

**Figure 2:**
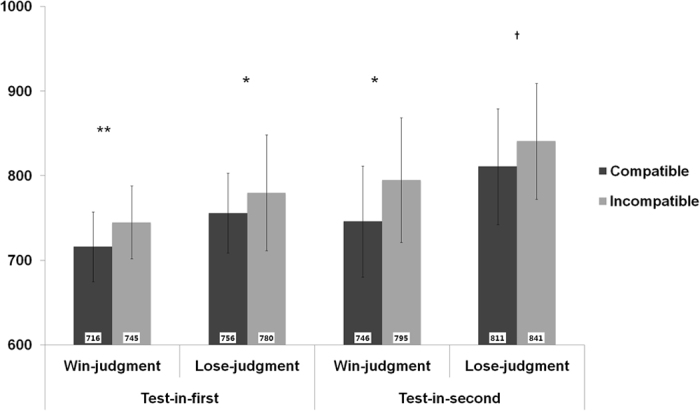
Response times (ms) in Experiment 1. The error bars represent one standard error of the mean, and asterisks or cross represent significant or marginally significant differences between two corresponding conditions (^**^p < 0.01, *p < 0.05, ^**†**^0.05 < *p* < 0.1).

For the analysis of RTs (see Fig. 2), the three-way ANOVA revealed significant main effects of Judgment, *F* (1, 16) = 12.33, *p* = 0.003, *η*_*p*_^*2*^ = 0.44, and Compatibility, *F* (1, 16) = 20.39, *p* < 0.001, *η*_*p*_^*2*^ = 0.56. Bonferroni-corrected post-hoc contrasts confirmed that the mean RT for Win-judgment trials (*M* = 750.52 ms, 95% confidence interval [CI] [648.61, 852.42]) was much shorter than that for Lose-judgment trials (*M* = 796.98 ms, 95% CI [684.35, 909.61]), and that the mean RT for Compatible trials (*M* = 757.02 ms, 95% CI [656.57, 857.47]) was shorter than that for Incompatible trials (*M* = 790.48 ms, 95% CI [677.74, 903.22]). No significant main effect of Target Position was found, *F* (1, 16) = 0.68, *p* > 0.250, *η*_*p*_^*2*^ = 0.04. Furthermore, neither the two-way nor the three-way interaction effects were significant *p*s > 0.250. Combined with accuracy analyses, we can say that the RT effects were not the result of a speed-accuracy trade-off. In line with the hypothesis, the main effect of Compatibility confirmed the schematization of large winner and small loser.

## Experiment 2

Experiment 1 demonstrated that size information indeed interfered with winning and losing judgments. To form a closed circle between mental representations of competition outcomes and size, Experiment 2 sought to confirm the reverse of our findings in Experiment 1: namely, the influence of competition outcome on size estimation. If this were true, a winning gesture would be estimated as larger than would a losing gesture.

## Methods

### Participants

A new group of 34 graduate and undergraduate students (15 females; mean age 22 years) were paid to participate in this experiment. The participants provided their written informed consent before the experiment, and were all rewarded with course credits or ¥20. All experimental procedures were approved by the Research Ethics Board of Zhejiang University, and were performed in accordance with the approved guidelines.

The power analysis indicated that a sample size of 34 subjects was required for the study to have 80% power to detect a medium-sized effect size (dz = 0.5) at a two-sided alpha level of 0.05; thus, we stopped collecting data when we reached this number of subjects.

### Stimuli

The same series of gesture pictures in Experiment 1 were adopted in Experiment 2. In the memory array, two gestures occupied the same rectangular area, which ranged from 4.7° × 4.0° to 7.5° × 6.5°, and were centred 6° to the left and right of a central fixation cross. The to-be-adjusted size of the probe gesture ranged from 2.8° × 2.4° to 9.5° × 8.2°. The other aspects of the stimuli were the same as in Experiment 1.

### Design and Procedure

**Figure 3:**
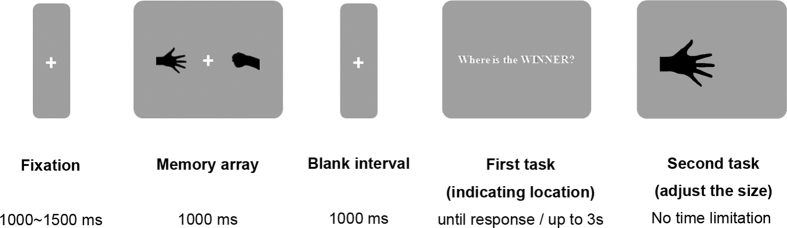
An example of the procedure in Experiment 2.

The full experimental procedure is shown in Fig. 3. This experiment comprised two tasks. In the first task, each trial began with a central fixation cross presented randomly for a duration of 1,000–1,500 ms. Then, two different gestures were displayed as the memory array for 1,000 ms. Participants were asked to memorize the gestures and their actual sizes. Then, after a 1000-ms blank interval, an instruction appeared on the screen requiring participants to indicate the location of the target gesture (the winner or the loser) in the memory array, by pressing key ‘z’ or ‘x’ to indicate on the left or right. Once the participant had responded correctly, a probe gesture with a to-be-adjusted size was presented in the same location as the previously indicated gesture, signalling the start of the second task. The to-be-adjusted size of the probe gesture was equally likely to be bigger or smaller than its actual size. Participant were asked to adjust the size of this probe gesture by using the keyboard (‘↑’ to increase the diagonal by 0.1° and ‘↓’ to decrease by the same amount) until the probe gesture matched what they remembered was the actual size of the target gesture. They then indicated that they had adjusted it to the appropriate size by pressing the space bar, which locked in the final value and began the next trial. Participants were informed that accuracy was most important and that they could manipulate the size as long as necessary to achieve an accurate estimate.

The only independent variable in this experiment was the role of the indicated gesture in the memory array (i.e., Winner condition or Loser condition). We recorded the final sizes, and adopted the relative size difference, calculated as (final size − actual size)/actual size, as the dependent variable. Dependent variable values above or below 0 represented a larger or smaller adjusted size than the actual size, respectively.

Each participant completed 96 trials in each condition, resulting in a total of 192 trials. The winner–loser situation in the memory array (i.e., Rock–Scissors, Scissors–Paper, and Paper–Rock) and the position of the test gesture were counterbalanced in each condition. The whole experiment was divided into six sessions, each separated with a 2-minute break. Before the formal experiment, there were at least 15 practice trials to ensure that the participants understood the instructions.

## Results

The mean accuracy of the first task of all participants was 96.26%, thus ensuring that there were enough valid trials for further analysis in the second task. The relative size differences of these conditions in the second task were compared using a paired-sample *t*-test, which found that they significantly differed, *M*_*winner*_ = 2.04%, *M*_*loser*_ = −0.16%, *t*(33) = 6.93, *p* < 0.001, 95% CI [1.55%, 2.84%]. This salient difference revealed that compared to defeat, victory would lead a size overestimation of a corresponding gesture.

## General Discussion

According to the above experiments, we found that the competition outcome can influence size estimation of the gestures involved and vice versa. Specifically, the results in Experiment 1 indicated that faster responses were obtained in the compatible condition, where the winning gesture was larger than the losing one, compared to the incompatible condition, where the losing gesture was larger than the winning one. This coincides with the main hypothesis, wherein we proposed that size information could interfere with judgments about the outcome of a competition, even when the outcome is not contingent on body size. In contrast, in Experiment 2, the winning gesture was estimated as much larger than the losing gesture in the adjustment task we devised, demonstrating that there is a reciprocal influence of competition outcome on size estimation. These findings provide evidence for an embodied view of cognition, according to which abstract concepts are encoded as accumulated bodily experiences of concrete sensorimotor information^19^,^20^,^21^.

As noted in the introduction, the association of size information and competition outcome is likely to have both phylogenetic and ontogenetic bases. Large-bodied animals, with their associated strength and muscularity, were more likely to be the winners of physical fights and competition for resources. Having a solid basis in the evolutionary process, this combination of large physical size and high probability of victory is further reinforced in the social experience across humans’ ontogenetic course, thus consolidating the representation in our cognitive system. Hence, size information was able to become a stable dimension for which to predict victory and defeat. Interestingly, this connection appears to be stable enough that it is activated in situations that are not determined by physical size or strength (e.g., the RPS game). Thus, despite the increasingly complicated nature of competitions and determinants of their outcomes, size information, being a primitive factor, appears to have remained as an embodied concept of the distinction between victory and defeat. This is likely to underlie the reason for the big–winner and small–loser association observed in the current study. Our results are consistent with those of Holbrook and colleagues’ study^12^, which proposed that two systems—an antecedent formidability representation system and a derived system representing other attributes that are intimately associated with competition—coexist in the mind. We provided supportive evidence for this notion—namely, new traits are derived and modified from an existing ancestral trait by reusing neural pathways through the phylogenetic process of descent, while the ancestral trait is preserved without losing its original functions. In this way, both the derived and ancestral ones overlap in the brain^12^,^22^,^23^.

One may argue that social power is a predominant aspect of the relationship between competition outcome and size, since previous research has shown that social power and size have an analogic association^24^,^25^. Winners may be thought of as having greater capability than losers, and, in some situations, will obtain greater resources and higher social status as the spoils of their victory. In this case, besides social power, factors such as one’s own ability might be connected to the concept of winner. Indeed, our results are not likely completely explained by this social power hypothesis. Social power is typically conceptualized as a relatively stable state over a prolonged period, whereas the role of each contestant in the RPS game changes constantly. Although an individual presenting Rock might win against Scissors, they may be defeated in another round against Paper. Altogether, the concepts of victory and defeat are quite distinct from social power.

There was an unexpected finding in examining the main effect of Judgment in further detail: there was a general tendency for participants to perform a Win judgment faster than a Lose judgment. It is interesting to speculate on the psychological mechanism underlying this judgment bias. From an embodiment perspective, one plausible reason is that mental representations for winners are relatively larger than are those for losers, making the win-related mental information salient in the mind and easy to judge.

This study has some limitations. First, the current results cannot clarify the more complicated issue of whether such concept representations influence perception itself 26,27 or various other post-perceptual phases (e.g., maintenance and response)^12^,^28^. We speculate that the distortion of estimated size occurs in the post-perceptual phase rather than in perceptual phase, since general-purpose perceptual representations are superior to specific-purpose representations in guiding responses, and thus any perceptual bias that facilitates performance in a certain task could potentially impair performance in other types of task. Additional evidence is needed to achieve a more nuanced perspective. Second, we used winning and losing gestures to represent victory and defeat, respectively, while concealing other parts of the contestant’s body. To enhance the external validity of our study, some major body parts should be added to the probe stimuli. We expect that the influence of competition outcome on size estimation of gestures would extend to the contestant’s entire body, even when victory or defeat is determined only by the hands. Third, our experimental design restricted participants as bystanders of competition; we cannot say whether the reciprocal association is found when they are contestants themselves.

Taken together, our findings demonstrated a closed circle between mental representations of competition outcome and size. Furthermore, given that the RPS game’s final outcome cannot be predicted by size information, it is reasonable to infer that this two-way interference is a result of unconscious metaphorical thinking.

## Additional Information

**How to cite this article**: Yu, W. *et al*. Humans Conceptualize Victory and Defeat in Body Size. *Sci. Rep.* **7**, 44136; doi: 10.1038/srep44136 (2017).

**Publisher's note:** Springer Nature remains neutral with regard to jurisdictional claims in published maps and institutional affiliations.
